# A Man

**Published:** 1858-07

**Authors:** 


### A MAN

WILL give any woman a seat in a public conveyance, rather than allow her to stand five minutes; for he does it, not to the mistress or the maid, to beauty or to wrinkles, but he does it to womankind, to the representative of his mother, his sister, his wife, his daughter; for the person standing before him bears one or more of these relations to some one: and what man's heart is there, that would not warm up in gratitude to another, even to a hod-carrier, who should promptly and cheerfully yield his seat to *his* mother, or wife, or daughter. Besides, very few of our women can stand in a moving vehicle, without positive inconvenience and very possible danger. The male who allows it may read a paper or book most vigorously, and may be intently looking through the window in another direction at—nothing; but it won't do; he feels mean all the time, and every additional moment, his better nature scourges him more and more.

